# Trial Interviews to Explore Glycogen Storage Disease Type Ia Patient Experiences Following Gene Therapy

**DOI:** 10.36469/001c.155666

**Published:** 2026-02-12

**Authors:** Diane M. Turner-Bowker, Jessica Butler, Shayna Egan, David A. Weinstein, David F. Rodriguez-Buritica, Ayesha Ahmad, María-Luz Couce, Rebecca Riba-Wolman, John J. Mitchell, Christina Theodore-Oklota

**Affiliations:** 1 Ultragenyx Pharmaceutical, Inc., Novato, California, USA; 2 Ultragenyx Pharmaceutical Inc., Novato, California, USA; 3 Lumanity, San Francisco, California, USA; 4 University of Connecticut, Farmington, Connecticut, USA; 5 University of Texas McGovern Medical School, Houston, Texas, USA; 6 University of Michigan, Ann Arbor, Michigan, USA; 7 University Clinical Hospital of Santiago de Compostela, IDIS, CIBERER, Santiago de Compostela, Spain; 8 Montréal Children’s Hospital, Montréal, Québec, Canada

**Keywords:** glycogen storage disease type Ia, glycogen storage disease, health-related quality of life, qualitative interviews, exit interviews, gene therapy

## Abstract

**Background:**

Glycogen storage disease type Ia (GSDIa) is a rare, inherited, autosomal recessive deficiency of glucose-6-phosphatase (G6Pase), an enzyme necessary in glycogenolysis and gluconeogenesis. To maintain normal blood glucose levels and ensure survival, individuals living with GSDIa must frequently consume complex carbohydrates (eg, uncooked cornstarch). Dietary management can result in chronic complications and significant patient burden. DTX401 (pariglasgene brecaparvovec) is an investigational adeno-associated virus serotype 8 vector (AAV8)–based gene therapy designed to restore endogenous glucose production.

**Objectives:**

Patient experience interviews were conducted as part of an open-label, phase 1/2 dose-escalation trial (NCT03517085) evaluating the safety and efficacy of DTX401 in adults ≥18 years with GSDIa.

**Methods:**

Telephone interviews were conducted at Weeks 24, 52, and 104, using a semistructured interview guide. Qualitative interview data were audio recorded, transcribed, coded, and analyzed.

**Results:**

Most (86%; n = 6/7) reported overall symptom improvement and reduced burden following DTX401 treatment. Three (43%) reported no negative outcomes following gene therapy; 4 (57%) mentioned at least one negative change attributed to instances of blood sugar instability, lifestyle, or diet adjustments. Satisfaction fluctuated across timepoints; however, most were somewhat satisfied/very satisfied with gene therapy at Weeks 24 (80%), 52 (86%), and 104 (86%). No participants reported being very dissatisfied.

**Discussion:**

Following DTX401 treatment, most participants reported substantial reduction in cornstarch intake and corresponding improvements in symptoms, physical function, diet management, emotional function, self-perception, social function, sleep quality, work performance, and overall health. Few negative changes were reported. While some results regarding met expectations were mixed, most indicated they would still want gene therapy even if they had to continue cornstarch and if they had continued diet restrictions, and most reported satisfaction with treatment. While the study had limitations, interview results suggest that DTX401 helps to address aspects of the condition and treatment that patients have identified as burdensome.

**Conclusions:**

Most interviewees in this open-label trial of investigational DTX401 described positive experiences, including substantial reduction in burden and improved health-related quality of life following treatment throughout the trial. To optimize patient outcomes and experience with gene therapy, guidance on and close monitoring of dietary changes during implementation should be provided.

## INTRODUCTION

Glycogen storage disease type Ia (GSDIa) is a rare inherited disease due to an inborn error of carbohydrate metabolism caused by variants in the *G6PC* gene.^1^ *G6PC* encodes glucose-6-phosphatase (G6Pase),^1^ an enzyme critical for glucose homeostasis. GSDIa causes a deficiency of G6Pase, minimizing the ability to release or generate glucose from the liver. Patients are at risk for severe hypoglycemia and metabolic abnormalities (eg, excess glycogen storage; production of lactic acid, uric acid, alanine, and triglycerides).^2–4^ Early diagnosis and treatment are essential for minimizing long-term complications.

There are no approved pharmacologic therapies targeting the underlying cause of GSDIa. Current management involves a regimented, medically prescribed diet typically requiring uncooked cornstarch every 3 to 6 hours, with or without a high-amylopectin waxy maize starch (Glycosade®), scheduled meals high in complex carbohydrates, and avoidance of nonutilizable sugars and fasting. Cornstarch and Glycosade® are digested slowly to help stabilize blood glucose when taken as scheduled.^5^ Although uncooked cornstarch has been the mainstay of GSDIa glucose management for decades, it requires lifelong patient and family commitment, negatively impacts quality of life, and can lead to wide fluctuations in blood glucose levels, suboptimal metabolic control, and chronic complications.^6,7^ Missing a single dose may cause severe hypoglycemia with lactic acidosis and even death.

There is a substantial humanistic burden associated with GSDIa. Patients experience hypoglycemic events, despite best efforts toward condition management.^8^ Adherence to a strict treatment regimen is challenging. Cornstarch can be unpalatable and cause gastrointestinal symptoms. There is constant fear that missing or delaying a single dose could result in a severe hypoglycemic event. Frequent cornstarch consumption interrupts daily activities and patients and caregivers describe a variety of negative physical, emotional, and psychosocial consequences.^8–12^

DTX401 (pariglasgene brecaparvovec) is an investigational adeno-associated virus serotype 8 vector (AAV8)–based gene therapy designed to deliver the human wild-type *G6PC* transgene to hepatocytes and restore endogenous glucose production. In a phase 1/2, open-label, safety and dose-finding study of DTX401 in adults with GSDIa (NCT03517085), participants in all cohorts showed significant cornstarch reductions from baseline to Week 52. DTX401 treatment was well-tolerated and had an acceptable and manageable safety profile.^13^

As part of the DTX401 phase 1/2 study protocol,^13^ qualitative interviews were conducted with trial participants at study Weeks 24, 52, and 104 to explore their clinical trial experience, any changes in GSDIa-specific health-related quality of life (HRQoL) following DTX401 treatment, and perspectives on gene therapy in relation to existing standard-of-care treatment.

## METHODS

### Sample

Twelve adults (≥18 years) with GSDIa recruited from clinical sites in the United States (US), Canada, the Netherlands, and Spain participated in *A Phase 1/2, Open-Label Safety and Dose-Finding Study of Adeno-Associated Virus (AAV) Serotype 8 (AAV8)-Mediated Gene Transfer of Glucose-6-Phosphatase (G6Pase) in Adults with Glycogen Storage Disease Type Ia (GSDIa)* (NCT03517085, NCT03970278).^13^ While qualitative interviews were planned after the start of the trial, they were intended to be conducted as part of the trial protocol (ie, embedded interviews). The interview inclusion criteria included individuals participating in the phase 1/2 trial and there were no exclusion criteria. Because the interviews were planned as part of a protocol amendment after the trial had already started, only a subset of trial participants were able to be recruited for interviews; this included only those who had not yet passed their Week 24 or Week 52 study visit by the time ethics approval was received. Clinical site staff contacted trial participants to identify and schedule possible interview dates as close as possible to their Week 24, Week 52, and Week 104 study visits. All direct communication with participants was conducted by the clinical site staff. Lumanity (formerly known as Endpoint Outcomes) researchers provided a conference dial-in to clinical sites for participants to use at the time of their scheduled interview.

### Materials

Semistructured interview guides (key questions shown in **Supplementary Table S1**) were used to explore patient perspectives on GSDIa, gene therapy, exogenous glucose intake, diet, and treatment satisfaction. Specifically, the Week 24/Week 52 and Week 104 interview guides included topics, questions, and probes designed to explore the effect gene therapy had on symptoms and impacts of GSDIa, the effect gene therapy had on diet and treatment (eg, cornstarch intake), and satisfaction with treatment. Week 52 and Week 104 interviews followed up on changes to cornstarch intake based on responses provided during the prior interviews.

### Procedure

Following ethics approval, 30-minute telephone interviews were conducted with each participant by Lumanity researchers who have expertise in patient-centered outcomes research via web-based teleconference at or around Weeks 24, 52, and 104. Interviews were audio-recorded with participant consent; and data were transcribed, anonymized, coded using version 8.0 or higher of ATLAS.ti, and analyzed.^14,15^ Each transcript was coded (ie, codes were applied to specific text within each transcript) by one of four Lumanity researchers, and a research supervisor addressed any discrepancies or questions. All coders received comprehensive instruction in established qualitative research methodologies, including grounded theory and the constant comparative method. In addition, each coder completed specialized training in coding procedures tailored to concept elicitation and trial interviews, as well as training in the use of ATLAS.ti, the qualitative analysis software employed during the project.

The coding process (codebook sample shown in **Supplementary Table S2**) was guided by established qualitative research methods, including grounded theory and constant comparative method. Specific grounded theory methods applied to this research included collecting and analyzing data in parallel (ie, initiating coding of transcripts while interviews are ongoing), letting the coding scheme be dictated by the data and not preconceived notions, constantly comparing and contrasting concepts to inform relationships among the data (ie, constant comparative method), and using memos within individual transcripts as necessary to explain findings and inform the next step of analysis (ie, aggregating transcripts and harmonizing codes).^14,15^ Frequencies of concepts were reported, with accompanying exemplary quotes. Thematic saturation of concept was not evaluated; data collected from all available trial participants was reported.

## RESULTS

### Sample Characteristics

While the trial sample included 12 participants, trial interviews were implemented after the trial had already started. No baseline interviews were conducted. Also, some participants (Cohort 1, n = 3; Cohort 2, n = 1) had already passed their Week 24 or Week 52 study visit; thus, interviews were not conducted with those participants. One Cohort 2 participant was interviewed but not recorded due to ethics restrictions. Thus, the analysis sample included 7 patients (n = 5 at Week 24, n = 7 at Weeks 52 and 104) interviewed between October 2019 and November 2022. The sample mean age at baseline was 27.4 years; 43% were female (**Table 1**).

**Table 1.** Sample Characteristics

**Phase 1/2 Trial Cohort^a^ (N=12)**		**Interview Sample by Timepoint, n**		
**Week 24^b^**	**Week 52**	**Week 104**		
Cohort 1 (n = 3)	2.0×1012 GC/kg with reactive steroid regimen; 6 weeks, at starting dose of 40 mg/day, after ALT elevation	0	0	0
Cohort 2 (n = 3)	6.0×1012 GC/kg with reactive steroid regimen; 6 weeks, at starting dose of 40 mg/day, after ALT elevation	0	1	1
Cohort 3 (n = 3)	6.0×1012 GC/kg with optimized reactive steroid regimen; 7 weeks, at starting dose of 60 mg/day, after ALT elevation	2	3	3
Cohort 4 (n = 3)	6.0×1012 GC/kg with prophylactic steroid regimen; 8 weeks, at starting dose of 60 mg/day, starting on Day 1	3	3	3
**Interview Sample Demographic Information (N=7)**		**n (%)**		
Gender				
Female		3 (43)		
Male		4 (57)		
Mean age (range), years		27.4 (18-42)		
Ethnicity, n (%)				
Non-Hispanic		7 (100)		
Race, n (%)				
White		7 (100)		
Region, n (%)				
US		3 (43)		
Canada		1 (14)		
Spain		1 (14)		
The Netherlands		2 (29)		

Abbreviations: ALT, alanine aminotransferase; GC, genome copies.

^a^Some trial participants were not interviewed because the interview research started after the trial was underway. Note that the interview analysis sample includes 7 of the 12 trial participants.

^b^Five participated in Week 24 interviews and all 7 participated at Weeks 52 and 104.

### Changes in Signs/Symptoms, Functional Impacts, and HRQoL

Across interview timepoints, most participants (86%, n = 6/7) primarily reported positive and meaningful changes in GSDIa following DTX401 treatment, although 1 participant (14%) reported no change in GSDIa. Three (43%, n = 3/7) reported no negative changes in HRQoL following DTX401 treatment, and 4 (57%, n = 4/7) reported at least one negative change, primarily at Week 104. Reduction in cornstarch intake was identified as a top improvement at both Weeks 52 and 104.

**Figure 1.**
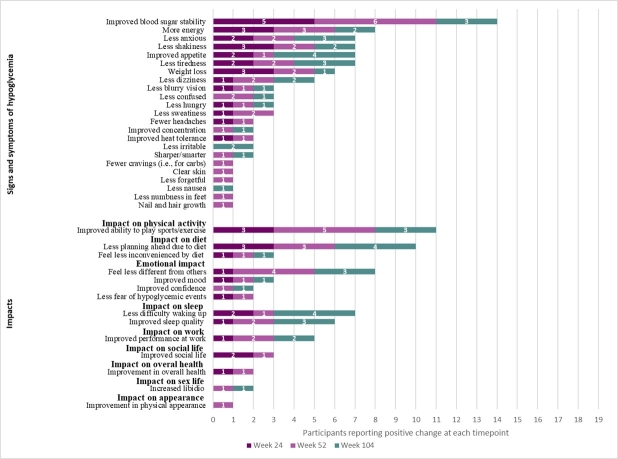
Trial Interview Participant Reports of Sign, Symptom, and Impact Improvements Following DTX401 Gene Therapy

**Signs/symptoms:** Across interview timepoints, participants most commonly reported improved blood sugar stability, more energy, less anxiety, less shakiness, improved appetite, less tiredness, weight loss, and less dizziness (**Figure 1**).

During Week 24 interviews, participants most commonly reported improved blood sugar stability (100%, n = 5/5), less shakiness (60%, n = 3/5), more energy (60%, n = 3/5), and weight loss (60%, n = 3/5). Participants described improved blood sugar stability both in terms of no longer having lows and having stable blood sugar.

During Week 52 interviews, participants most commonly reported improved blood sugar stability (86%, n = 6/7) and more energy (43%, n = 3/7). A few new changes were reported at Week 52 by participants that were not reported during Week 24. Two participants (29%) described less confusion; and clear skin, less forgetful, better concentration, mentally sharper, increased confidence, improved appearance of skin, nails, and hair, improved libido, reduced cravings, and less numbness in feet were each mentioned by 14% (n=1/7) of participants.

During Week 104 interviews, participants commonly reported improved appetite (57%, n = 4/7), blood sugar stability (43%, n = 3/7), less anxiety (43%, n = 3/7), and less tiredness (43%, n = 3/7). New changes reported during Week 104 included less irritability (29%, n = 2/7) and less nausea (14%, n = 1/7).

**Figure 2.**
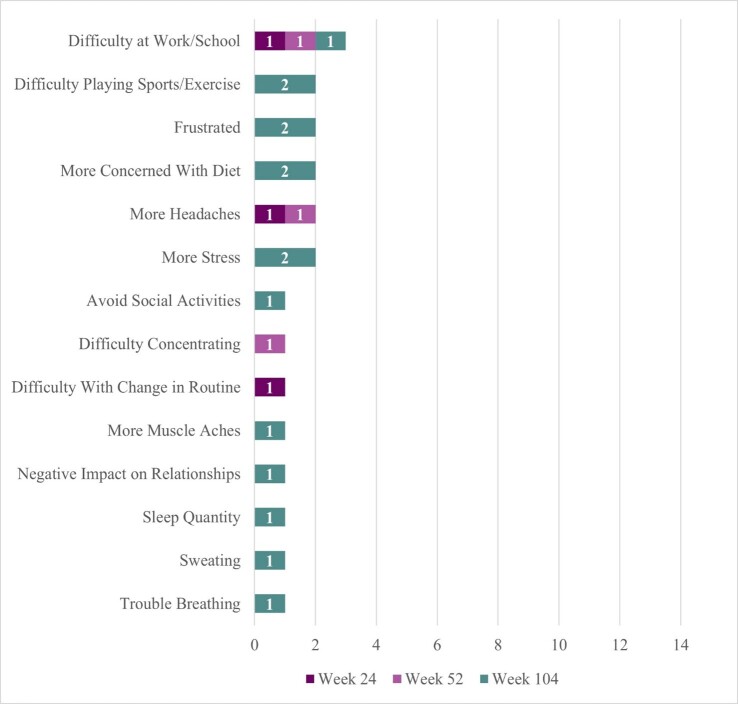
Trial Interview Participant Reports of Negative Changes Following DTX401 Gene Therapy

Two participants reported some negative sign/symptom changes (**Figure 2**). One participant reported headache at Week 24, headache and difficulty concentrating at Week 52, and frustration, stress, sweating, heavier breathing when blood sugar is high, and muscle ache (although no longer reporting frequent headaches or difficulty concentrating) at Week 104. This participant described life-changing benefits from DTX401 treatment (“it’s like a new life…a new beginning”) but shared that condition management can be stressful because “there is not an exact formula on how to handle [their] health now.” They reported that they had improvements in concentration and focus at Week 104 because they no longer needed to constantly “look at the clock” to manage their GSDIa. This same participant attributed the muscle ache to gout; explained that sweating was due to greater sensitivity to heat at Week 104 rather than instances of low blood sugar; and that they breathe heavier when their blood sugar levels are high (which they do not like experiencing, but interpreted positively as a signal that they need to take action to lower their blood sugar level).

The second participant reported no negative symptoms at Week 24 and Week 52 but described experiencing frustration and stress at Week 104. This participant explained that while their cornstarch intake was lower, they could “last longer” until the next dose, and eat more of their desired foods (eg, spaghetti), they experienced some frustration and stress due to instances of low blood sugar (though not as severe/low as prior to treatment) and changes to their diet management, requiring them to adapt their lifestyle at Week 104.

**Table 2A** presents sample quotes from interview participants describing changes in signs/symptoms following DTX401 treatment.

**Table 2.** DTX401 Trial Interview Sample Quotes

**A. Signs/Symptoms**
Improved *My blood sugar doesn’t crash as bad…it’s not as life-threatening. I have some time to fix it myself before it gets scary. (Week 24)* *And I feel very balanced. … I’ve not experienced hypoglycemia in the way I’ve used to. My blood sugars are consistent and do not drop the way they did before gene therapy. … These consistent blood sugars, I can execute my day without having to worry about highs and lows during the day. I would get stomachaches, headaches, exhaustion, all from high and low blood sugar. I do not have those now. (Week 24)* *I used to basically want to sleep [during the day] as much as I could and now I’m good on like six hours’ sleep most days…I’m much less tired overall … I don’t want to sleep all day. (Week 104)* Worsened/mixed *I think I’m a lot happier – I think with the gene therapy, only because…my blood sugar doesn’t affect my mood as much…I’ve gotten more stressed out, though, since the gene therapy, probably, which has been a negative effect … It’s harder to manage. It’s not like an exact formula on how to handle my health now…Like before the gene therapy, I was super-stable, even though my health was more critical. It was more critical that I pay attention. I was super-good at paying attention, so I was super-stable. But now, it’s not like cut to a perfect T on how to manage this. So, it definitely stresses me out sometimes, only because it’s very changing – it’s always changing. (Week 104)* *Because of a lot of changes in the diet, I had to adapt it, for it. It gave me more stress. (Week 104)*
**B. Functional Impacts**
Improved *Yeah, I don’t have to stop and snack as much. I feel better. My body feels better. When I exercised before the gene therapy, I’d feel really run down or tired. I didn’t want to do it for long. But now, I’m ready to keep going – like could go forever … the activities that my friends want to do sometimes – I’m able to do them now because of it. (Week 104)* *…now I don’t have to wake up in the middle of the night to take any treatment, so that’s a huge improve my life, because now I can sleep eight hours in a row. (Week 104)* *My diet is so much less cornstarch now, I can enjoy other foods, I can participate in meals and really enjoy the calories I’ve taken instead of having them completely dominated by cornstarch. (Week 24)* *…previously anytime I would leave the house, I would have to have multiple cornstarch doses with me. I would have to ensure that I watched my clock and had many, many alarms set, but now that I’m not having as many doses during the day, I don’t have to do any of those things, and it definitely has reduced the level of stress and monitoring. (Week 104)* *It’s just made me better at what I do for a career…. I can handle it so much better… (Week 52)* Worsened/mixed *When I’m not doing enough cornstarch, I would have to eat more, and definitely is a lot of fine-tuning, which… definitely takes away from work. I think I feel probably more different than I have only because I used to have a routine… with the gene therapy, I have to do a lot more. (Week 24)*
**C. Overall HRQoL**
*I would just say this [experience in the gene therapy clinical trial] is the single most wonderful change in my health in my lifetime. I would do it again in a moment without question, and it has absolutely and completely changed my life for the better. (Week 24)* *I’m just able to live more life…I feel more like normal people. (Week 104)* *Well, less blood sugar fluctuations mean better health and so less health problems…It just kind of made my life better overall…I feel like I’m not going to die early anymore and – yeah, just better life…I feel like it’s just reversing the death sentence. (Week 104)* *…I have to do many tests and overall it didn’t change very much my life. (Week 52)* *I feel so much better about myself… Like not have to live by the clock. (Week 52)* *I feel safer now… have more freedom and can do more things than I could before the therapy. (Week 104)*
**D. Top Improvements**
Reduction or elimination of cornstarch *Yes, this is such a big deal because I went three days without doing cornstarch except for Glycosade®, and literally I was crying happy tears because I couldn’t bel – I lasted so long or I was – even live a day without my cornstarch. It was unbelievable. … If this was a change, this would be number one. (Week 24)* *Yes, that is absolutely a change, not just that I no longer have to drink cornstarch. It makes a big difference, that I no longer need to weigh it and take it with me if I go somewhere. It makes it feel a little safer to go without because, for example, now I’ll just eat a sandwich instead and that’s obviously available on every street corner. And cornstarch is not, so you can simply go out without taking a bag full of cornstarch with you. (Week 24)* Energy *I’ve had more energy and more consistent blood sugars during the day. … Having more energy means I’m more able to participate in work and family, that’s with friends, and just participate in life. (Week 104)* *… a better sleeping schedule, too. And more energy due to that. … It was a big change, too, because I used to be really tired all day, so, yeah. (Week 24)* Blood sugar stability *… the gene therapy helped my body to be able to control my blood sugar. (Week 52)* *My blood sugar used to be very volatile. Now it’s very stable. Well, much more stable than it was. … less blood sugar fluctuations mean better health and so less health problems. (Week 104)*
**E. Willingness to Have DTX401 Gene Therapy Even with Continued Diet Restrictions**
*Oh, yeah. I think the gene therapy was great…Just all the benefits you talked about. It just all around makes you a healthier person. (Week 52)* *Yes … Oh, because the study treatment has reduced the amount of – amount and frequency of cornstarch I have to take… (Week 104)* *No, I don’t think so, because you participate in something like this to see and gain progress. (Week 24)*
**F. Willingness to Have DTX401 Gene Therapy Even with Continued Cornstarch**
*Yeah, sure, because it’s an improvement. It’s not a cure, but it’s an improvement. So yeah, I like to have the gene therapy. (Week 52)* *Yes, if it was a reduced amount of cornstarch, a significantly reduced amount of cornstarch. No if it wasn’t. (Week 104)* *If it had worked better, I would do it … (Week 104)*
**G. Likes/Dislikes of Cornstarch Treatment**
*It’s just a lot of cornstarch. You kind of feel bloated all the time. (Week 52)* *It is extra calories, it is carbohydrates that I may not want. It is heavy to drink before bed. It is an imposition. It is in no way is driven by my desires. It is to keep myself alive. (Week 104)* *I don’t have a problem with it, because it’s giving me safety. (Week 104)* *During the night it’s quite easy for the cornstarch, because it’s quite hard to eat at night, but then during the day it’s easier if, if you can just have normal meals, cause then you get also the nutrients and it makes make you feel fitter and stronger. (Week 52)*
**H. Likes/Dislikes of DTX401 Gene Therapy**
*Yeah. [I like] reduced cornstarch doses, reduced cornstarch amount, reduced hypoglycemia. (Week 104)* *It is a lot of monitoring… Because it is a diet-based condition and treatment, it is much more sort of intrusive in daily life in terms of monitoring and details. (Week 104)* *… I don’t need to be as strict with my lifestyle as I used to. I don’t need to watch the clock all the time, I am more flexible. (Week 24)* *It’s harder to take the control of your glucose now that you have the gene therapy… its always changing… it’s making improvements with every step… that means that what makes my glucose steady now is not going to be the same in a month. (Week 52)*
**I. Treatment Expectations**
*Definitely to be more stable, my – blood sugar-wise. I was expecting my blood sugar to be more stable, and I was definitely expecting, like if I was to come off cornstarch, to stay off cornstarch. (Week 104)* *I hoped to remove most or all cornstarch. I hoped to really open up my diet in terms of what I could eat and I hoped to be able to eat based on my own hunger or interest versus blood sugar maintenance. (Week 104)* *Well, I – maybe I have higher expectations, because I thought that it would be a cure, maybe not having to take any cornstarch or …, or not having hypoglycemia never. But now I see that it’s improvement, but not a cure, so yeah, my expectations were higher. (Week 52)*
**J. Treatment Satisfaction**
*I feel safer now… I have more freedom and can do more things than I could before the therapy. (Week 104)* *The fact that [cornstarch] is no longer a distraction from my day and having to plan for five different doses during the day... I have more kind of appetite for actual food… Now I can go out for dinner, I can go out with a friend, I can go for a walk or to the drugstore and not have to worry about bringing anything with me and that has been a very positive experience. (Week 52)* *It’s very stable. It didn’t do any wrong or good... it didn’t change very much my life. (Week 52)* *My health is better… It’s just my blood sugar. Before I had better control. But because it’s like a gene therapy, like it’s still like trial and error… [Although] I feel so much better about myself… Like not have to live by the clock. (Week 52)*

**Functional impacts:** Across interview timepoints, participants most notably reported improved physical activity (eg, increased ability to play sports/exercise), diet (eg, less planning ahead for diet), emotional function (eg, feeling less different from others), and sleep (eg, less difficulty waking up, better sleep quality) (**Figure 1**).

During Week 24 interviews, participants reported improved ability to play sports/exercise (60%, n = 3/5) and less planning ahead due to diet (60%, n = 3/5). Participants described improved physical stamina, increased energy that enabled improved physical function, greater flexibility in diet, and less fear of fluctuating blood sugar levels, reducing worry and improving participation in daily life routines.

During Week 52 interviews, participants most commonly reported improved ability to play sports/exercise (71%, n = 5/7), feeling less different than others (57%, n = 4/7), and less planning ahead due to diet (43%, n = 3/7).

During Week 104 interviews, participants reported improved appetite, less difficulty waking up, and less planning ahead due to diet, which were each reported by 4 participants (57%, n = 4/7). Further, some participants described that they were able to have more flexibility in their diet, felt more refreshed upon awakening, and experienced longer fasting intervals.

Four participants reported some negative changes in functional impact (**Figure 2**). The same participant who reported headache and difficulty concentrating symptoms at earlier trial timepoints reported more difficulty at work/school at Week 24 (20%, n = 1/5) which persisted for this same participant at Weeks 52 and 104, and attributed this difficulty to changes in their blood sugar levels and the “fine tuning” required to “fix” them, noting that they need to stop what they are doing to address them (eg, “snack consistently”). This same participant reported difficulty with change in routine at Week 24 (attributed to the need to manage a changing diet regimen following gene therapy), and more concern with diet, negative impact on relationship with family and friends (more focus on condition management and less time for relationships), and sleep quantity (although sleep quality improved) at Week 104. One participant described more concern with their diet at Week 104. This is the same participant who reported overall positive outcomes from treatment yet increased frustration and stress symptoms at Week 104, attributable to adaptations needed for diet management following treatment. Finally, 2 participants reported difficulty in sports/exercise at Week 104 despite improvement reported in this concept at Weeks 24 and 52. At Week 104, both reported that their ability to play sports/exercise was about the same as it was prior to receiving treatment. One participant posited that the improvement experienced at earlier timepoints may have been affected by other medicines (eg, corticosteroids).

**Table 2B** presents sample quotes from interview participants describing changes in their functional impacts following DTX401 treatment.

**Overall HRQoL**: Most participants described overall HRQoL improvements at Week 24 (80%, n = 4/5), Week 52 (86%, n = 6/7), and Week 104 (71%, n = 5/7); 1 participant reported no improvement in overall HRQoL across timepoints (**Table 2C**).

**Top improvements**: Across interview timepoints, the concepts most frequently identified as top improvements included reduction in cornstarch intake, improved energy levels, and improved blood sugar stability (**Table 2D**).

### Patient Perspectives on Study Treatment

Most participants with evaluable data at Week 24 (75%, n = 3/4), Week 52 (67%, n = 4/6), and Week 104 (57%, n = 4/7) reported they would still want gene therapy even if they had to adhere to a restricted diet (**Table 2E**). Similarly, most participants at Week 24 (100%, n = 5 of 5), Week 52 (100%, n = 7/7), and Week 104 (71%, n = 5/7) said they would still want gene therapy treatment even if they had to continue cornstarch (**Table 2F**).

**Perspectives on taking cornstarch**: Four participants each reported liking one aspect of cornstarch at Weeks 24, 52, and 104, with reasons including not having to eat as much when taking cornstarch, taking before bedtime helped them sleep through the night, helping them feel stable/safe, and helping them fix a problem with their blood sugar (**Table 2G**). Three participants, across all interview timepoints, reported not liking any aspect of taking cornstarch. Participants commonly described disliking the inconvenience of the regimen, bloating, high calorie and carbohydrate count, and bad taste (**Table 2G**).

**Perspectives on taking DTX401 gene therapy:** When asked what they liked about taking DTX401, across timepoints, participants most commonly described the efficiency of the therapy as a one-time painless infusion, the potential for improvement, reduced need for cornstarch, and greater freedom in life (**Table 2H**).

When asked what they disliked about taking DTX401 study treatment, across timepoints, participants most commonly described that they disliked taking steroids, the trial visit frequency, or that there was nothing they disliked about DTX401 gene therapy (**Table 2H**).

### Treatment Expectations and Satisfaction with Treatment

**Treatment expectations:** The most commonly reported treatment expectations across timepoints included reduction or elimination of cornstarch, improved blood sugar stability, and more flexible diet. Other expectations included improved ability to play sports/exercise, weight loss, more energy, and improved overall health (**Table 2I**).

**Figure 3.**
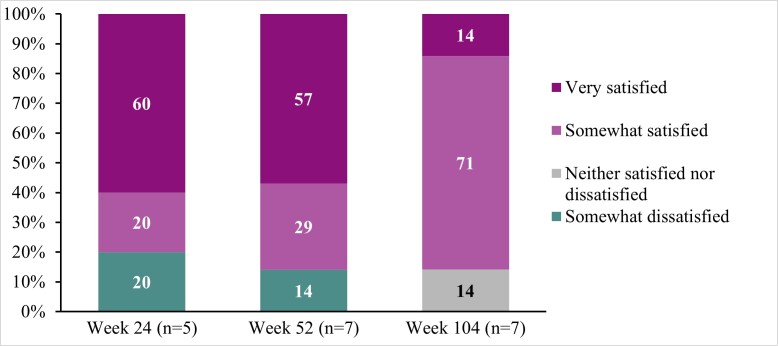
Satisfaction with DTX401 Gene Therapy Across Interview Timepoints Treatment satisfaction was assessed using a 5-point response scale, ranging from “very satisfied” to “very dissatisfied.”

**Treatment satisfaction:** While participant reports of satisfaction fluctuated across interview timepoints, most were somewhat satisfied or very satisfied with DTX401 gene therapy at Weeks 24 (80%), 52 (86%), and 104 (86%), and no participants reported being very dissatisfied at any interview timepoint (**Figure 3; Table 2J**).

## DISCUSSION

During trial interviews, most DTX401-treated participants reported substantial reduction in cornstarch intake, aligning with reported trial results.^13^ Interview participants perceived corresponding improvements in HRQoL, including improved symptoms, physical function, diet management, emotional function, self-perception, social function, sleep quality, work performance, and overall health. Across interview timepoints, participants most consistently reported improved blood sugar stability, improved ability to play sports/exercise, less planning ahead due to diet, less shakiness, more energy, weight loss, and improved sleep quality as positive changes. Top improvements reported by participants across interview timepoints included reduction in cornstarch intake, improved energy levels, and improved blood sugar stability. Overall, few negative changes were reported, most of which were at Week 104. Findings suggest that DTX401 helps to address aspects of the condition and its standard of care treatment that patients have identified as burdensome.^8,16–18^

While there were some mixed results regarding whether trial expectations were fully met, most participants preferred gene therapy over standard-of-care treatment, most liked the efficacy of gene therapy as a one-time infusion, and most indicated that they would want gene therapy even if they had to continue cornstarch and even if they still had diet restrictions.

Most reported satisfaction with treatment across interview timepoints, although some fluctuations were reported. No participants reported being very dissatisfied with treatment at any of the timepoints. Trial procedures and tests that may have influenced satisfaction ratings could be addressed in future research through patient education to clarify trial requirements.

The following opportunities for improvement were identified: (1) preparation and patient support for the dietary changes required by transitioning from cornstarch as a major source of calories to a more balanced diet and (2) review of glycemic control data with patients to facilitate shared decision-making on nutritional adjustments and reduce fear of hypoglycemia when cornstarch doses are reduced.

This interview study had several limitations. One major limitation was a very small sample size and a demographically homogeneous sample (7/12 participants, all White), driven by sampling from a small, phase 1/2 ultra-rare disease trial population. An important strength of this interview study is the longitudinal design, which captures the participant experience over multiple trial timepoints. However, because the interview research started after the phase 1/2 trial was underway, only a subset of trial participants (n = 7/12) were interviewed, and no baseline interviews were possible. Baseline interviews to capture burden prior to trial treatment could help to limit potential bias when assessing the experience of change over time at the participant level. Quantitative results from the phase 1/2 trial have been published,^13^ and this interview research yields important insights into the patient experience of DTX401 treatment to support the interpretation of quantitative outcomes. Future research of this nature could be improved using a mixed-methods approach that integrates the qualitative and quantitative analysis. Another consideration is that this research describes interview participants’ perception of their trial experience without formal testing of relationships among variables. While four trained researchers with expertise in patient interview research coded transcripts over the course of this lengthy study and efforts were made to ensure consistency in the coding approach (eg, any questions/uncertainties/discrepancies reviewed by a supervising researcher), the coding process was limited in that only one interview researcher coded each transcript and coding confirmation (and interrater reliability) was not assessed as part of the process. Additionally, interview conduct was contingent on trial visits proceeding as intended during the COVID-19 pandemic. Finally, a longer interview duration would have provided more time for additional in-depth probing questions.

## CONCLUSION

Most participants interviewed during an open-label, phase 1/2 dose-escalation trial (NCT03517085) evaluating the safety and efficacy of DTX401 in adults at least 18 years of age with GSDIa described positive experiences, including reduced treatment burden, improved HRQoL, and satisfaction with treatment. Results from an open-label trial may be exploratory, be subject to potential bias, and have limited generalizability. Nevertheless, these qualitative trial interviews provide important insights into the patient-reported experience of trial participation, potential for this investigative treatment, and support for the interpretation of quantitative trial outcomes. Results suggest that to optimize outcomes and patient experience with gene therapy for GSDIa, anticipatory guidance on dietary changes and the need for close monitoring during implementation of these changes should be provided. Findings from the ongoing phase 3 randomized, double-blind, placebo-controlled study of investigative DTX401 (NCT05139316) will further inform the GSDIa patient experience of treatment.

### Ethics Approval and Patient Consent Statement

The protocol, including embedded trial interviews, was approved by the institutional review board at each participating center. All procedures followed were in accordance with the ethical standards of the responsible committee on human experimentation (institutional and national) and with the Helsinki Declaration of 1975, as revised in 2000. Informed consent was obtained from all patients prior to participation in the study.

### Meeting Presentation

Components of this research were summarized in a poster presentation at the Society for Inherited Metabolic Disorders (SIMD) 45th Annual Meeting, Charlotte, North Carolina, April 14-17, 2024.

## Supplementary Material

Online Supplementary Material
