# Supplementary material for: Trial Interviews to Explore Glycogen Storage Disease Type Ia Patient Experiences Following Gene Therapy
Source: J Health Econ Outcomes Res. 2026 Feb 12;13(1):39–47. doi: 10.36469/001c.155666 (PMC12906305; doi:10.36469/001c.155666)
Supplement: Online Supplementary Material [file jheor_2026_13_1_155666_330733.pdf]

Journal of Health Economics and Outcomes Research

Online Supplementary Material

Trial Interviews to Explore Glycogen Storage Disease Type Ia Patient Experiences Following Gene Therapy. *JHEOR*. 2026;13(1):39-47. doi:10.36469/jheor.2026.155666

Table S1: Interview Guide Outline

Table S2: Example Codes

This supplementary material has been provided by the authors to give readers additional information about their work.

This is an open-access article distributed under the terms of the Creative Commons Attribution 4.0 International License (CCBY-4.0). View this license's legal deed at http://creativecommons.org/licenses/by/4.0 and legal code at http://creativecommons.org/licenses/by/4.0/legalcode for more information.

Table S1. Interview Guide Outline

**Section of Interview Guide**	**Specific Interview Guide Topics**
Effect of study treatment	• Changes experienced due to study treatment<br>• Direction of change (positive or negative)<br>• Type of change (eg frequency, severity, duration)<br>• Meaningfulness of change<br>• Top 3 improvements since starting clinical trial<br>• Changes or lack of changes since prior interviews (weeks 52 and 104 only)
Effect on diet and treatment	• Daytime cornstarch intake (frequency and amount)[a]<br>• Daytime cornstarch intake prior to trial<br>• Daytime cornstarch intake at time of interview<br>• Change in daytime cornstarch intake<br>• Meaningfulness of change in daytime cornstarch intake<br>• Nighttime cornstarch intake (frequency and amount)[a]<br>• Nighttime cornstarch intake prior to trial<br>• Nighttime cornstarch intake at time of interview<br>• Change in nighttime cornstarch intake<br>• Meaningfulness of change in nighttime cornstarch intake<br>• Ease of maintaining prescribed diet<br>• Ease of tracking prescribed diet<br>• Likes and dislikes about cornstarch compared to study treatment<br>• Willingness to use treatment while maintaining restricted diet<br>• Willingness to use treatment while continuing to take cornstarch
Treatment satisfaction	• Treatment expectations<br>• Extent to which expectations were met/not met<br>• Likes and dislikes about treatment<br>• Overall treatment satisfaction rating<br>• Changes in satisfaction over the course of the clinical trial<br>• Experience filling out questionnaires daily<br>• Any other feedback

[a]Participants were asked about cornstarch intake in two separate steps: first about frequency, then about amount. For clarity, the topics are presented in this table in a combined format.

Table S2. Example Codes

Concept	Code	Example Related Interview Guide Question/Data
Treatment effect	Tx effect: [description of change]: [Positive change/Negative change]	• Did you experience any changes due to the study treatment?<br>• What was the change like (eg, was it positive or negative)?
	Tx effect: [description of change]: Frequency improvement	Did the frequency or amount of [symptom/impact] improve?
	Tx effect: [description of change]: Meaningful: [Yes/No/Missing]	Was this change meaningful to you?<br>• [*If yes*:] Why was it meaningful?
Daytime cornstarch frequency	Diet and tx effect: Cornstarch: Times taken during day previously: [No. of times]	Prior to starting the clinical study, how many times did you take corn starch during the day *[define "day" for the participant]:* from the time you wake up to the time you go to bed)?[a]
	Diet and tx effect: Cornstarch: Times taken during day now: [No change/{No. of times}]	Now, after about *[length of time]* weeks in the study, about how many times do you take corn starch during the day?
	Diet and tx effect: Cornstarch: Times taken during day now: Change: [Meaningful yes/ meaningful no]<br>Diet and tx effect: Cornstarch: Times taken during day now: Change: Meaningful no desired change: [No. of times]	[*If the number of times they take corn starch during the day has changed*:] Is this change meaningful to you?<br>• [*If yes:*] Why is it meaningful?<br>• [*If no:*] What would be a meaningful improvement in the number of times you take corn starch during the day?
Effect on diet	Diet and tx effect: Maintaining prescribed diet: [Easy/Difficult/Missing]	Did you find it easy or difficult to maintain your prescribed diet? Why?
	Diet and tx effect: Keep track of prescribed diet: [Easy/Difficult/Missing]	Did you find it easy or difficult to keep track of your prescribed diet? Why?
Treatment satisfaction	Tx satisfaction: Expectation: [description]	What did you expect from the treatment?
	Tx satisfaction: Expectation met: [Yes/No/ Missing]	Have your treatment expectations been met?<br>• [*If yes*:] Please explain how.<br>• [*If no:*] Why not?
	Tx satisfaction: Study tx likes: [description]	What did you like about the treatment? Why?
	Tx satisfaction: Study tx dislikes: [description]	What did you dislike about the treatment? Why?
	Tx satisfaction: Satisfaction changed since start of study: [Yes: {description}/No/ Missing]	How does your satisfaction with treatment now compare to when you started the clinical study?
Study experience	Tx satisfaction: Experience completing questionnaires: [Easy/Difficult/Missing]	• How was your experience with filling out the questionnaires at home each day?<br>• Was it easy or difficult to fill out the questionnaires each day? Please explain.

[a]Interview question included in Week 24 interview guide. At Week 52 and Week 104, participants were provided with their Week 24 interview response as a reference point in responding to the question regarding change in cornstarch intake.
